# Dimethyl pyrazol-based nitrification inhibitors effect on nitrifying and denitrifying bacteria to mitigate N_2_O emission

**DOI:** 10.1038/s41598-017-14225-y

**Published:** 2017-10-23

**Authors:** Fernando Torralbo, Sergio Menéndez, Iskander Barrena, José M. Estavillo, Daniel Marino, Carmen González-Murua

**Affiliations:** 1 Department of Plant Biology and Ecology, University of the Basque Country (UPV/EHU), Bilbao, Spain; 2 Ikerbasque, Basque Foundation for Science, Bilbao, Spain

## Abstract

Nitrous oxide (N_2_O) emissions have been increasing as a result of intensive nitrogen (N) fertilisation. Soil nitrification and denitrification are the main sources of N_2_O, and the use of ammonium-based fertilisers combined with nitrification inhibitors (NIs) could be useful in mitigating N_2_O emissions from agricultural systems. In this work we looked at the N_2_O mitigation capacity of two dimethylpyrazol-based NIs, 3,4-dimethylpyrazole phosphate (DMPP) and 2-(N-3,4-dimethyl-1H-pyrazol-1-yl) succinic acid isomeric mixture (DMPSA), on soil nitrifying and denitrifying microbial populations under two contrasting soil water contents (40% and 80% soil water filled pore space; WFPS). Our results show that DMPP and DMPSA are equally efficient at reducing N_2_O emissions under 40% WFPS conditions by inhibiting bacterial ammonia oxidation. In contrast, at 80% WFPS DMPSA was less efficient than DMPP at reducing N_2_O emissions. Interestingly, at 80% WFPS, where lowered oxygen availability limits nitrification, both DMPP and DMPSA not only inhibited nitrification but also stimulated N_2_O reduction to molecular nitrogen (N_2_) via nitrous oxide reductase activity (Nos activity). Therefore, in this work we observed that DMP-based NIs stimulated the reduction of N_2_O to N_2_ by nitrous oxide reductase during the denitrification process.

## Introduction

Nitrous oxide (N_2_O) represents an important environmental threat due to its high global warming potential of 265–298 times greater than carbon dioxide (CO_2_) with a lifetime of 121 years, together with its involvement in the destruction of the ozone layer^1^. Moreover, its total global emissions to the atmosphere have increased 6% since 2005^1^. Soil, both natural and managed, is considered the primary source of N_2_O in global greenhouse gas budgets^2^. Furthermore, it has been estimated that the agricultural contribution to anthropogenic N_2_O emissions represents around 70–80%^1,3^. Autotrophic nitrification and heterotrophic denitrification are responsible for most of these emissions^4^. Under aerobic conditions, nitrification is driven by ammonia-oxidising bacteria (AOB) and archaea (AOA), which oxidise ammonia (NH_3_) into hydroxylamine (NH_2_OH) through the ammonia monoxygenase enzyme (AMO) encoded by the *amoA* gene^5^. During the nitrification process, N_2_O can be produced as a secondary product. Through nitrifiers denitrification N_2_O can be also emitted by the reduction of nitrite (NO_2_^−^) directly to nitric oxide (NO), N_2_O or molecular nitrogen (N_2_)^6^. However, although both nitrification and denitrification processes can occur in wet soils where there is limited oxygen (O_2_) availability, the main source of N_2_O is usually the denitrification of nitrate (NO_3_^−^) by denitrifying microbes^7^. The denitrification pathway consists of four sequential reactions initiated by NO_3_^−^ reduction and carried out by nitrate reductase (Nar, Nap), followed by nitrite reductase (Nir), nitric oxide reductase (Nor), and nitrous oxide reductase (Nos), leading to the generation of N_2_ as an end-product^8,9^.

In agriculture, the magnitude of N_2_O emissions depends greatly on both the application of nitrogen (N) fertilisers and the effect of edaphoclimatic conditions on microbial activity, including O_2_ levels as well as temperature, pH, and the soil carbon:nitrogen ratio^10,11^. Nitrification inhibitors (NIs) have been extensively applied to keep N available, in the form of ammonium, in the soil for longer periods while lessening NO_3_^−^ leaching and mitigating N_2_O gas emission^12^. In this sense, the use of NIs in conjunction with ammonium-based fertilisers has been proposed as an excellent strategy for reducing N_2_O emissions^13–15^. A great number of molecules with the capacity to inhibit nitrification have been identified^16,17^. At present, the commercialised dicyandiamide (DCD) and 3,4-dimethylpyrazole phosphate (DMPP) are the most widely used NIs. The mode of action of DCD and DMPP has not been completely elucidated, but both of them are supposed Cu-selective metal chelators that may remove this AMO co-factor, therefore avoiding the oxidation of ammonium (NH_4_^+^) to NO_2_^−17^. Several studies have demonstrated similar efficiency for DMPP and DCD in mitigating N_2_O emissions^12^. However, DMPP reduces the ecotoxicological problems related to DCD, as it is applied at approximately one-tenth lower concentration than DCD^18,19^. Besides, plant capacity to take up DCD has been reported^20,21^ and indeed, traces of DCD have been found in dairy products from cows grazing in grasslands fertilized with DCD^22^.

The persistence of NIs and their capacity to reduce the microbial oxidation of NH_4_^+^ to NO_2_^−^, thus mitigating N_2_O emissions, have been shown to be affected by soil conditions including soil temperature, moisture^23–25^ and pH^26,27^. A very recent development is the new DMP-based inhibitor 2-(N-3,4-dimethyl-1H-pyrazol-1-yl) succinic acid isomeric mixture (DMPSA). The use of pyrazole compounds as nitrification inhibitors have the disadvantage of the highly volatility of pyrazole rings. To confer more stability and reduce pyrazole ring volatility, DMPSA holds a succinic residue bonded to the pyrazole ring instead of the more instable phosphate of DMPP. Therefore, DMPSA is stable with other fertilizers such as calcium ammonium nitrate or diammonium phosphate that would not be able to use with nitrification inhibitors such as DMPP. Both DMPP and DMPSA are structurally very similar but it is not still clear if these inhibitors have the same mode of action and efficiency when targeting soil nitrifying organisms. In fact, there are almost no studies on DMPSA^28,29^. To our knowledge, only Huérfano *et al*.^28^ have compared DMPSA and DMPP in a wheat-field. These authors found that both inhibitors exhibited a similar N_2_O-emissions-reducing capacity while maintaining crop yield and quality.

It is accepted that the nitrification inhibition action of DCD and DMPP reduces nitrifying bacterial populations. This is generally observed as a reduction in *amoA* gene copy number in AOB, although the effect on AOA *amoA* is less evident^17^. It is also probable that NIs mitigate N_2_O emissions through indirectly limiting denitrification processes by decreasing the availability of NO_3_^−^^23,30,31^. Finally, in the framework of reducing N_2_O emissions from agriculture, the last denitrification step by Nos (encoded by *nosZ*) becomes crucially important since this is the only enzyme capable of reducing N_2_O to N_2_.

Most studies describing the *nosZ* gene copy number after the application of NIs are related to organic fertilisation, and there is no consensus on how the *nosZ* gene abundance is affected. For example, in laboratory incubation experiments, Florio *et al*.^30^ observed that the application of DMPP jointly with cattle effluent reduced *nosZ* gene abundance. Interestingly, Barrena *et al*.^25^ observed an effect of DMPP stimulating *nosZ* gene abundance under 80% water filled pore space (WFPS) conditions. Similarly, Di *et al*.^24^ showed that DCD stimulated *nosZ* gene abundance at 130% field capacity. Regarding DMPSA, until now there is no study describing how it affects soil microbial populations. Therefore a greater understanding of how these molecules modulate soil microbiota to reduce the negative effect associated with nitrogen fertilisation is crucial to optimise fertilisation management.

In this context, the main objectives of this work were to study the effectiveness of DMPSA compared to DMPP in mitigating N_2_O emissions, and to quantify the behaviour of nitrifying and denitrifying microbial populations under two contrasting soil water-content conditions (40% and 80% WFPS). Moreover, since NIs are highly efficient at reducing N_2_O emissions in soils under low oxygen availability; in this work, we also explored the hypothesis that denitrification could be directly affected by DMP-based inhibitors.

## Results

### DMPP and DMPSA reduced nitrous oxide emissions and ammonium oxidation under both WFPS conditions

**Figure 1:**
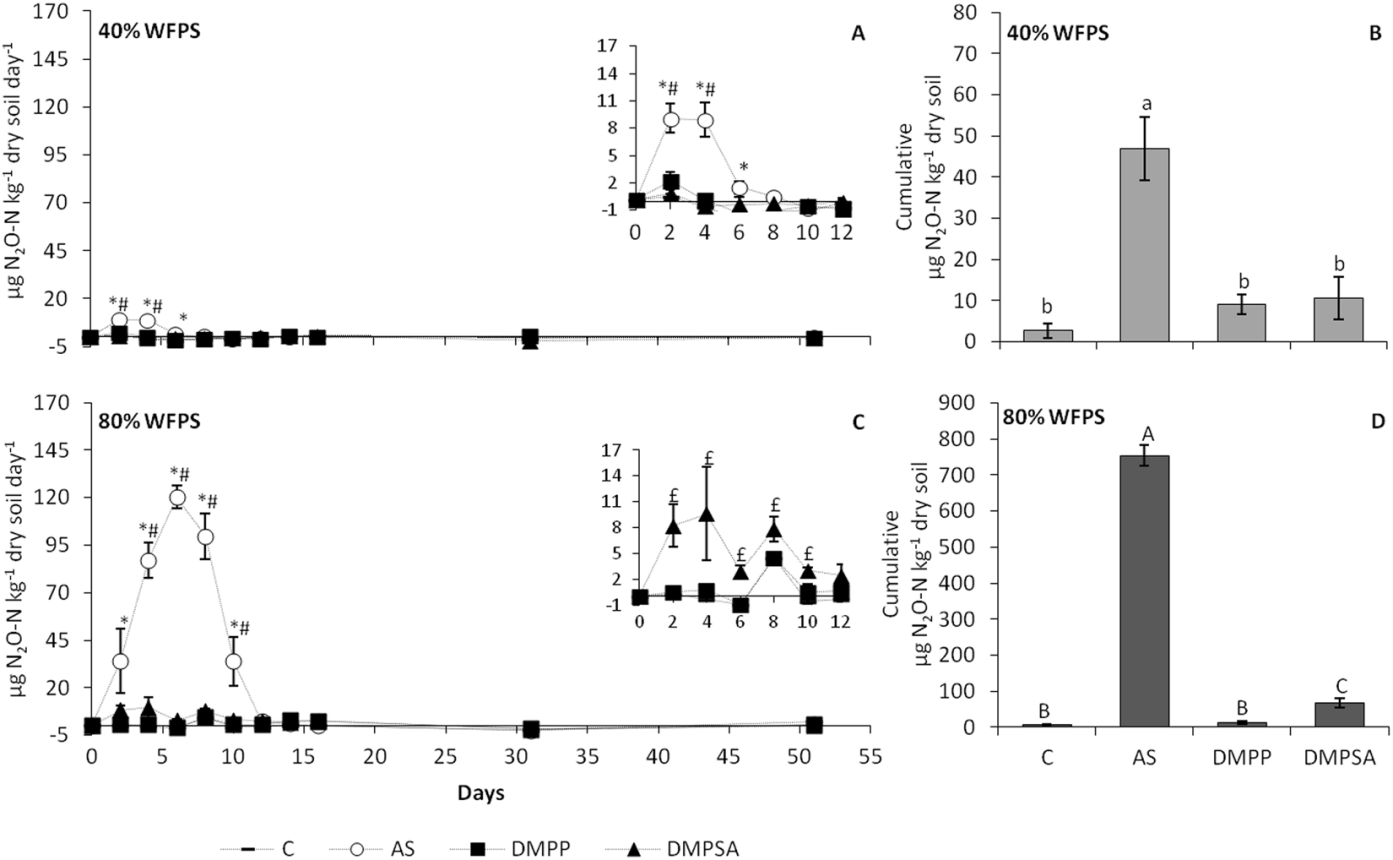
Daily (**A**,**C**) and cumulative (**B**,**D**) N_2_O emissions at 40% WFPS (**A**,**B**) and 80% WFPS (**C**,**D**) soil microcosms during the experiment. The inset graphs in sub-figures A and C show an amplified view of the daily N_2_O emissions for the first 12 days. For daily emissions, significant differences (p < 0.05) between DMPP and DMPSA with respect to AS are represented by * and #, respectively, and significant differences (p < 0.05) between DMPP with respect to DMPSA are represented by £. For cumulative emissions, significant differences (p < 0.05) are represented by different letters. Values represent the mean ± SE (n = 4). C = unfertilised control; AS = ammonium sulphate; DMPP = ammonium sulphate + DMPP; and DMPSA = ammonium sulphate + DMPSA.

**Figure 2:**
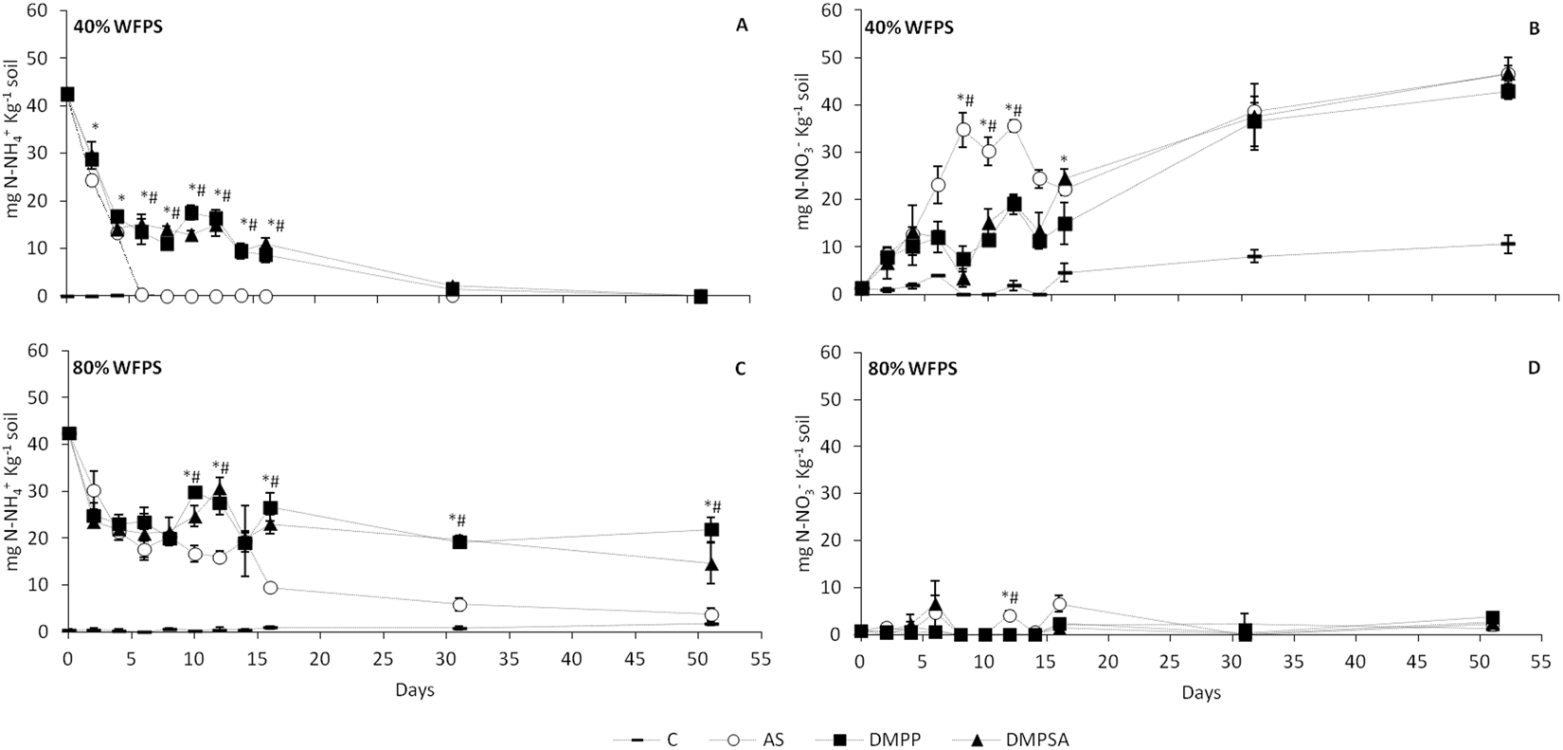
Evolution of soil ammonium (**A**,**C**) and nitrate content (**B**,**D**) at 40% WFPS (**A**,**B**) and 80% WFPS (**C**,**D**). Significant differences (p < 0.05) between DMPP and DMPSA with respect to AS are represented by * and #, respectively. Values represent mean ± SE (n = 3). Ammonium content for day 0 represents the total amount of NH_4_^+^ added to the samples.

Fertilisation with ammonium sulphate (AS) generated a clear N_2_O emissions peak during the first 12 days of incubation (Fig. 1). The magnitude of the N_2_O emitted was dependent on soil water content, since under 80% WFPS greater than ten times more N_2_O was emitted than at 40% WFPS (Fig. 1A,C). When NIs were applied together with AS, almost no N_2_O was emitted under either soil water content (Fig. 1). However, under 80% WFPS conditions, although both NIs reduced N_2_O emissions, in DMPSA-treated soils the cumulative N_2_O emission was significantly higher than in both the control and DMPP treatments; therefore, DMPP was more efficient at 80% WFPS (Fig. 1C,D). The unfertilised control treatments maintained low and constant values of both NH_4_^+^ and NO_3_^−^ regardless of soil WFPS. The higher nitrification rates expected under the more aerobic conditions (40% WFPS) provoked rapid oxidation of NH_4_^+^, which in AS-treated soils dropped to the level of the unfertilised-soil after six days of incubation (Fig. 2A). In contrast, the application of NIs led to a higher NH_4_^+^ content being maintained until day 16 (Fig. 2A). In agreement with the dynamics of NH_4_^+^ content, the level of NO_3_^−^ at 40% WFPS in AS-treated soils underwent a faster and more pronounced increase than in those with DMPP and DMPSA (Fig. 2B). At 80% WFPS, due to the limited oxygen availability that impairs nitrification, the NH_4_^+^ content stayed at relatively high levels until day 14 in all fertilised treatments; however, in the presence of NIs the higher NH_4_^+^ content was evident from day 10, and this was maintained until the end of the incubation period (Fig. 2C). Nitrate contents remained low throughout the entire experiment in all soils under 80% WFPS conditions (Fig. 2D).

### Expression and abundance of nitrification and denitrification genes

**Figure 3:**
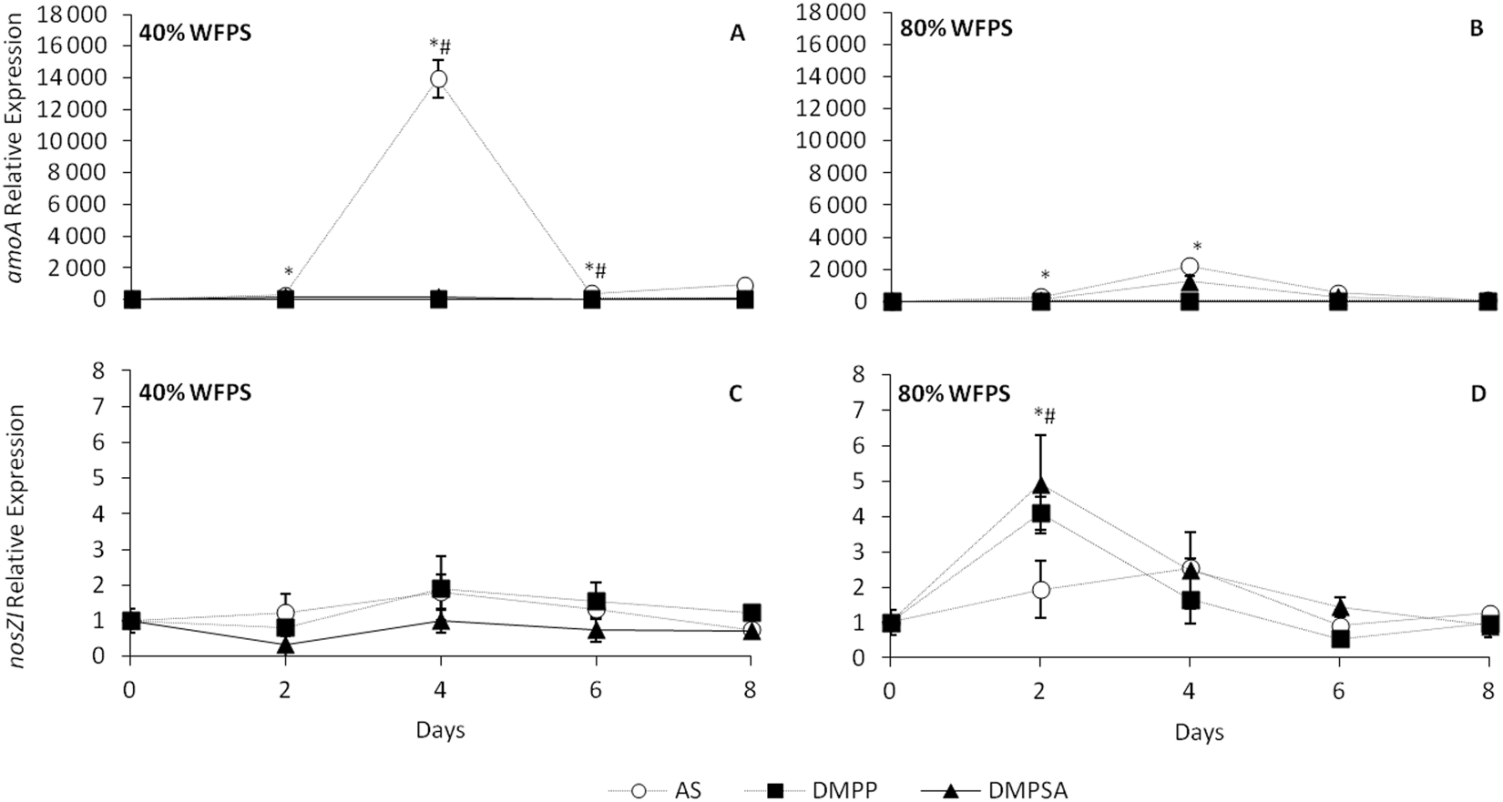
Relative expression of bacteria *amoA* **(A**,**B**) and *nosZI* (**C**,**D**) at 40% WFPS (**A**,**C**) and 80% WFPS (**B**,**D**) for the first 8 days. Significant differences (p < 0.05) between DMPP and DMPSA with respect to AS are represented by * and #, respectively. Values represent mean ± SE (n = 3).

To check how the different fertilisation regimes were affecting soil bacteria, we measured the expression of nitrifying and denitrifying genes in the first days of incubation. Under 40% WFPS conditions, bacterial *amoA* expression experienced a striking induction in AS-treated soils concomitant with N_2_O emissions, and this induction was completely blocked when DMPP or DMPSA were applied together with AS (Fig. 3A). Under 80% WFPS conditions, the magnitude of bacterial *amoA* expression in AS-treated soils was almost six times lower than with 40% WFPS on day 4 (Fig. 3A,B). DMPP also impeded *amoA* expression induction at 80% WFPS. In contrast, although the expression values recorded with DMPSA were not as high as when only AS was applied, the differences between these two treatments were not significant (Fig. 3B).

The expression of the denitrifying genes *narG*, *nirK* and *nirS* did not vary substantially, regardless of the fertilisation type (Supplementary Figure 2). Only *nirK* expression increased on day 4 after AS fertilisation at 40% WFPS (Supplementary Figure 2C). Interestingly, *nosZI* gene expression was induced 2 days from the onset of the incubation, when nitrification inhibitors were applied; this induction was exclusive to the 80% WFPS conditions, where denitrification is favoured due to low levels of O_2_ availability (Fig. 3D). The low intensity of the *nosZII* amplification signal meant we were unable to quantify its expression in any of the fertilisation regimes.

To confirm the results obtained through gene expression analysis, we also quantified the nitrifying and denitrifying abundances 16 and 51 days after fertilisation. The abundance of bacteria, quantified as 16*S rRNA* gene copy number per gram of dry soil, did not vary among the fertilised treatments at any of the incubation times (Supplementary Figure 3). The abundance of archaea did not vary between treatments at day 16 (Supplementary Figure 3C); however, at day 51 under 40% WFPS conditions, archaea abundance in AS-treated soils was lower than in the unfertilised control (Supplementary Figure 3D).

**Figure 4:**
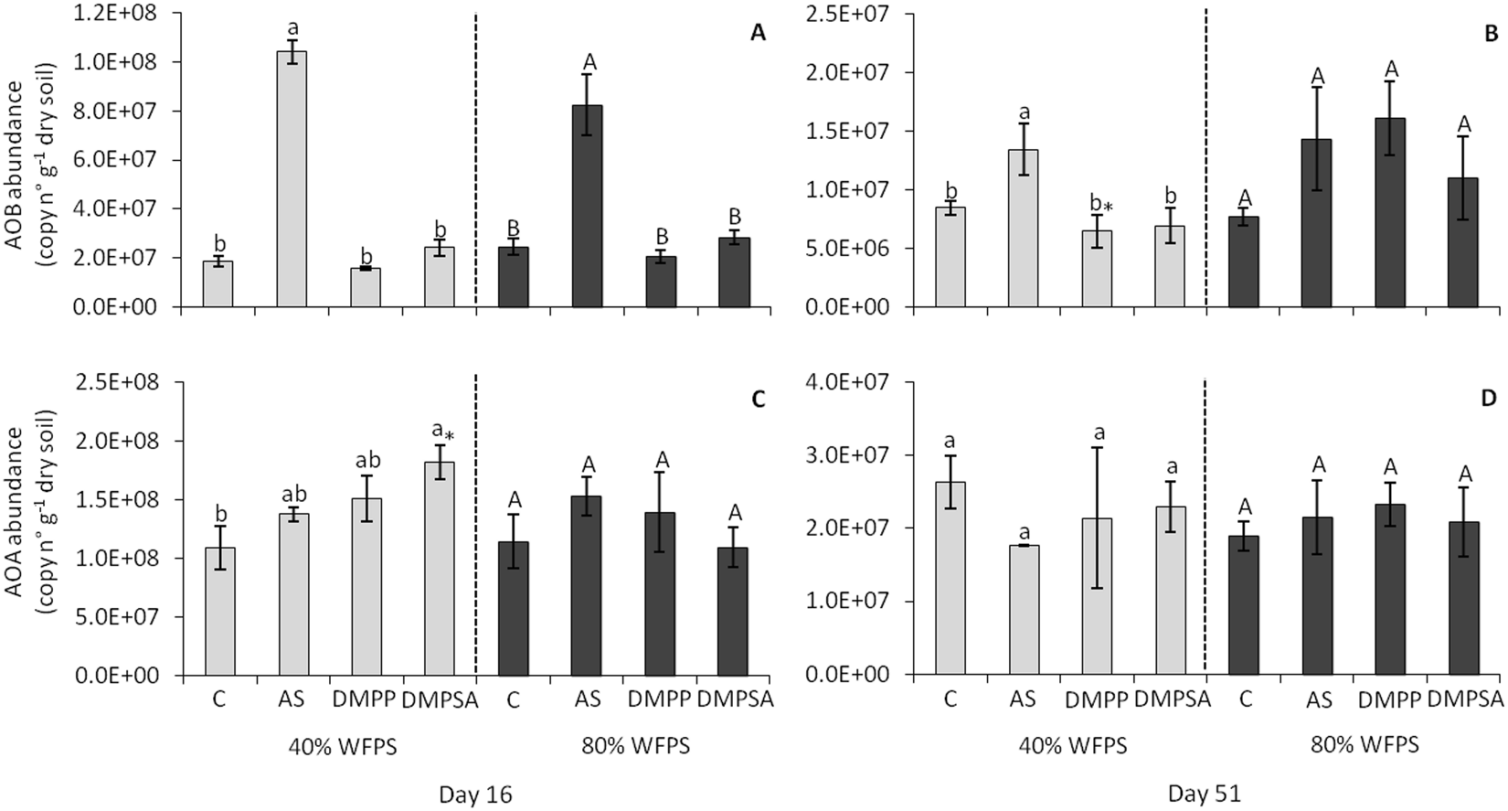
Abundance of AOB (**A**,**B**) and AOA (**C**,**D**) expressed respectively as bacteria and archaea *amoA* gene copy number per gram of dry soil at 40% WFPS (grey bars) and 80% WFPS (black bars), 16 (**A**,**C**) and 51 days (**B**,**D**) after treatment application. Significant differences (p < 0.05) between treatments within each WFPS condition are indicated with different letters. Asterisk (*) indicates significant WFPS effect for each fertilised treatment (p < 0.05). Values represent the mean ± SE (n = 4). C = unfertilised control; AS = ammonium sulphate; DMPP = ammonium sulphate + DMPP; and DMPSA = ammonium sulphate + DMPSA.

Nitrifying microbial abundances (AOB and AOA) were quantified by determining bacterial and archaeal *amoA* gene copy number per gram of dry soil. As shown in Fig. 4A, 16 days after fertilisation and regardless of soil WFPS, AS treatment stimulated the AOB population, which was around five times more abundant than in the unfertilised control. This stimulation was completely abolished when NIs were applied together with the fertiliser. Interestingly, the effect of AS on AOB dropped 51 days after fertilisation and was only evident at 40% WFPS (Fig. 4B). No differences were detected in AOA abundance among the fertilised treatments, regardless of WFPS and incubation time (Fig. 4C,D). The ratio of AOA gene copies over AOB gene copies (AOA/AOB) gives us an idea of the response of the community in the microcosm. AOA gene copies was not affected by the addition of AS. Nevertheless, NI-treated soils reduced AOB gene copy number, which resulted in a higher ratio AOA/AOB than in the soil treated only with AS (Supplementary Figure 4).

**Figure 5:**
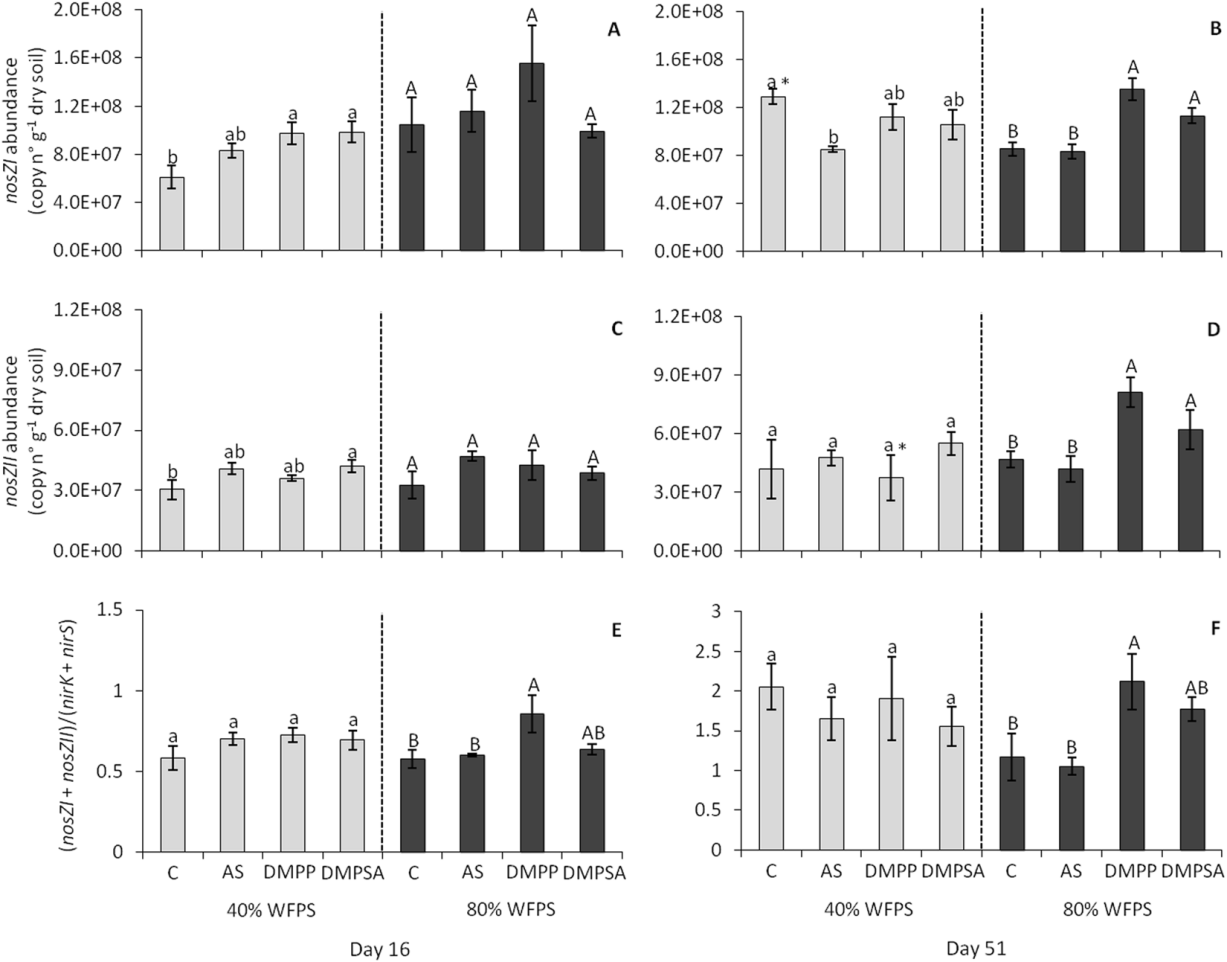
Abundance of *nosZI* (**A**,**B**), *nosZII* (**C**,**D**) expressed as gene copy number per gram of dry soil, and the ((*nosZI* + *nosZII*)/(*nirK* + *nirS*)) ratio (**E**,**F**) at 40% WFPS (grey bars) and 80% WFPS (black bars), 16 (**A**,**C**,**E**) and 51 days (**B**,**D**,**F**) after treatment application. Significant differences (p < 0.05) between treatments within each WFPS condition are indicated with different letters. Asterisk (*) indicates significant WFPS effect for each fertilised treatment (p < 0.05). Values represent the mean ± SE (n = 4). C = unfertilised control; AS = ammonium sulphate; DMPP = ammonium sulphate + DMPP; and DMPSA = ammonium sulphate + DMPSA.

Nitrate and nitrite-reducing bacteria were quantified by determining the copy number of *narG*, *nirK* and *nirS* genes per gram of dry soil. None of these gene abundances varied between the different treatments (Supplementary Figure 5A–F). The abundance of nitrous oxide-reducing bacteria was determined by quantifying the *nosZI* and *nosZII* gene copy number per gram of dry soil. As shown in Fig. 5, the *nosZ* gene copy numbers did not differ between the fertilised treatments at 40% WFPS. However, 51 days from the onset of the incubation at 80% WFPS, the abundance of both genes increased when DMPP or DMPSA were applied together with AS (Fig. 5B,D).

The ratio of the sum of *nosZI* and *nosZII* gene copies over the sum of *nirK* and *nirS* gene copies ((*nosZI* + *nosZII*)/(*nirK* + *nirS*)) gives us an idea of potential N_2_ *versus* N_2_O production; a higher ratio means a greater potential for N_2_O reduction^32,33^. At 80% WFPS, the ratio was higher with DMPP application compared to AS treatment, this difference being emphasised at day 51 (Fig. 5E,F). This fact suggests that although the potential for completing the denitrification pathway to N_2_ is enhanced in the presence of both NIs, DMPP is more effective than DMPSA at promoting the N_2_O reduction.

### DMPP and DMPSA induce nitrous oxide reductase activity under denitrifying conditions

**Figure 6:**
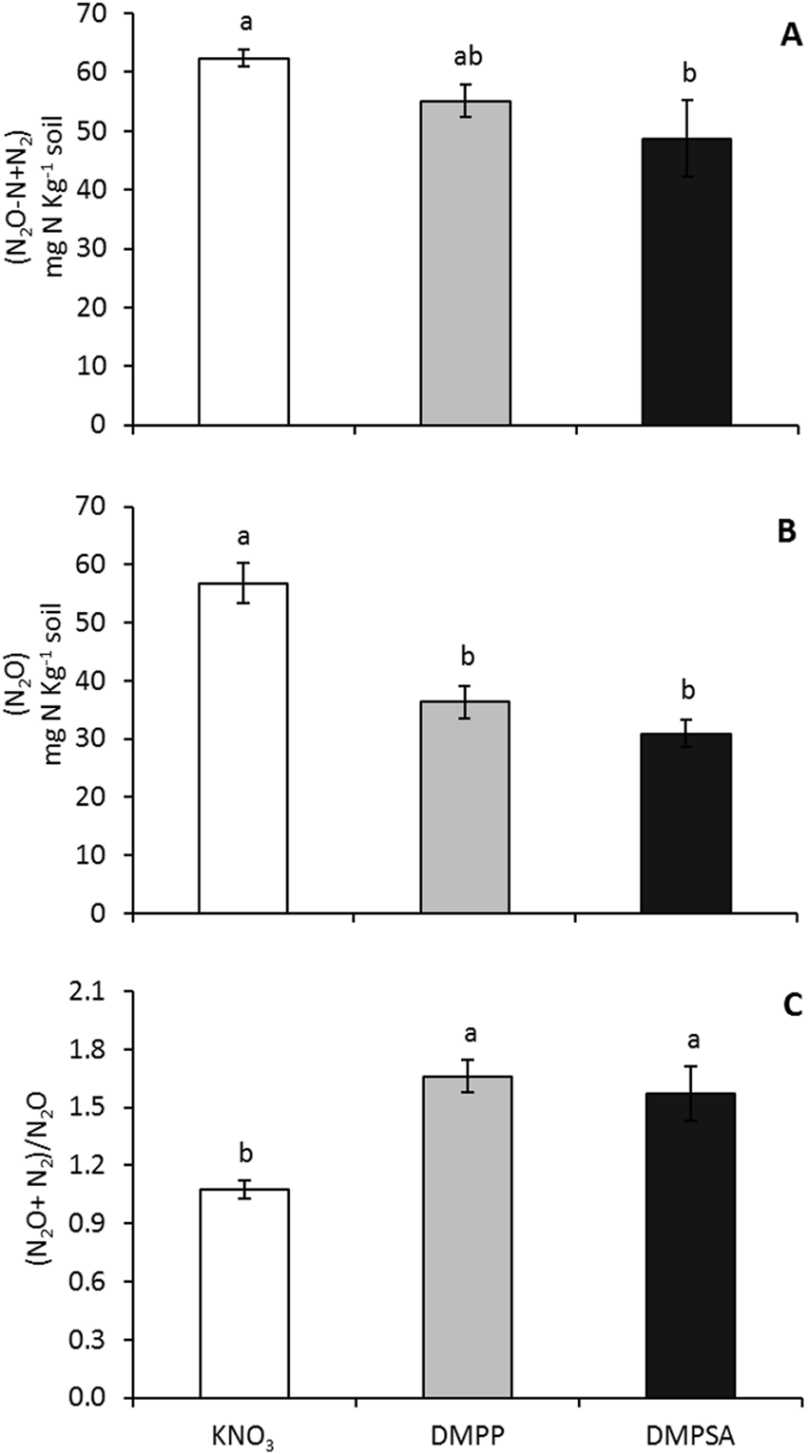
Denitrification activity up to N_2_O + N_2_ (**A**) (acetylene) or up to N_2_O (**B**) (non-acetylene) and nitrous oxide reductase activity (Nos activity) (**C**) expressed by the ratio of acetylene incubation over non-acetylene incubation ((N_2_O + N_2_)/N_2_O) in KNO_3_, KNO_3_ + DMPP, and KNO_3_ + DMPSA treatments. Significant differences (p > 0.05) are indicated with different letters. Values represent the mean ± SE (n = 4).

In order to confirm the effect of DMPP and DMPSA as potential inducers of N_2_O to N_2_ reduction under 80% WFPS conditions, we aimed to determine the activity of the denitrifying enzymes through a soil incubation experiment in denitrifying conditions after nitrate was added in a high concentration to induce the denitrification process. As shown in Fig. 6A, Nos activity was inhibited in acetylene-treated bottles; thus, higher N_2_O emissions were detected compared to non-acetylene-treated bottles. In acetylene-treated bottles DMPP had no effect respect to KNO_3_ control treatment. In contrast, DMPSA addition showed lower N_2_O emissions in acetylene-treated bottles compared to KNO_3_ control treatment (Fig. 6A). Interestingly, in non-acetylene-treated bottles, where Nos activity was active, both DMPP and DMPSA stimulated this activity reducing significantly N_2_O emissions (Fig. 6B). The ratio of acetylene-treated bottles over non-treated ones ((N_2_O + N_2_)/N_2_O) was higher when DMPP or DMPSA were applied jointly with KNO_3,_ supporting the hypothesis that these NIs induced the reduction of N_2_O to N_2_ (Fig. 6C). It should be noted that, although acetylene inhibition technique has received several criticisms, for instance because it does not completely inhibit the reduction of N_2_O to N_2_^34,35^, this method is useful for comparative purposes between treatments. In this sense, the absolute values should be taken with care.

## Discussion

NIs mode of action is not completely understood; however, it is generally accepted that their function is related to the inhibition of the AMO enzyme^16,17^. The effectiveness of NIs in reducing N_2_O emissions varies with land use, soil type, environmental conditions, and the type of fertiliser employed^12,36^. Indeed, NIs are also able to decrease N_2_O emissions under low O_2_ conditions, where the activity of nitrifying bacteria is limited and the main source of N_2_O is denitrification^23,24^.

Several studies have reported that the efficiency of DMPP in reducing N_2_O emissions is related to the inhibition of ammonium oxidation associated with AOB control^31,37,38^. In this work we also observed that DMPP reduced N_2_O emissions to the unfertilised control level (Fig. 1) concomitantly with ammonium oxidation inhibition (Fig. 2). This was further evidenced by the inhibition of AOB proliferation on day 16 (Fig. 4), and correlation analysis indicated that the cumulative N_2_O emissions (Fig. 1B) were positively correlated with the AOB abundance (r = 0.526, p < 0.05). Huérfano *et al*.^28^ observed the same N_2_O-emission-reducing behaviour of DMPP and DMPSA in a wheat field. Here we report a similar effect of both DMPP and DMPSA, observed under 40% WFPS conditions. Besides the commonly reported lower AOB population after NI application^24,31,37^, in this work we also found that both DMPP and DMPSA completely blocked the rapid induction of bacterial *amoA* gene expression provoked after fertilisation with AS (Fig. 3A,B). Similar results were also obtained recently when DMPP was added to soils amended with cattle effluent^30^, and plant residues^39^. This evidences the fact that NIs affect AOB, not only by inhibiting AMO activity^17^, but also by regulating *amoA* transcription. However, NIs were not observed to affect *amoA* from AOA as reported in previous studies^24,37,40^. Indeed, it has been suggested that the substantial cellular and genetic differences between AOB and AOA could explain the minor efficiency of NIs in targeting AOA^27,40^. Finally, as expected, gene expression levels and the gene copy number of denitrification pathway marker genes showed no significant variation caused by the use of NIs under 40% WFPS (Supplementary Figures 2, 5), in accordance with the specificity of nitrification inhibitors targeting AOB described by Kong *et al*.^41^.

When the available oxygen is limited, denitrification is the dominant force responsible for N_2_O production^36,42,43^. In our study, at 80% WFPS, the near lack of nitrate (Fig. 2D), accompanied by the huge increase in N_2_O emissions with respect to 40% WFPS conditions (Fig. 1), evidences that NO_3_^−^ consumption by denitrifiers is principally responsible for N_2_O emission. Nevertheless, nitrification does take place under low oxygen conditions, although at much lower rates^44–46^. In addition, although not completely understood, NIs have also been shown to efficiently mitigate N_2_O emissions under denitrifying conditions^23,25,47,48^. In our study, the stimulation of AOB abundance after AS application (Fig. 4A), together with *amoA* gene expression induction (Fig. 3B) and NH_4_^+^ content depletion through time (Fig. 2C), corroborates the presence of nitrifying activity at 80% WFPS, which provides the substrate for denitrification. However, it must be noticed that the decrease in NH_4_^+^ takes place much more slowly than at 40% WFPS (Fig. 2); moreover, *amoA* induction by AS fertilisation was around 6 times lower than at 40% WFPS, evidencing the expected lower nitrification rate under 80% WFPS conditions, where O_2_ availability is restricted. At 80% WFPS, both NIs reduced N_2_O emissions and inhibited nitrification, evidenced by the persistence of NH_4_^+^ in the soil (Fig. 2C), together with the decrease in the AOB population (Fig. 4A). Surprisingly, DMPSA proved to be less efficient than DMPP at reducing N_2_O emissions (Fig. 1D). Indeed, no significant *amoA* expression inhibition was observed with DMPSA (Fig. 3B). In view of the low level of nitrification induction observed after the application of AS at 80% WFPS, together with the significant efficiency of NIs in reducing N_2_O emissions, the effect of NIs on the denitrification process was analysed in order to corroborate our hypothesis that NIs could also be acting on denitrification.

We found that both DMPP and DMPSA stimulated the expression of the *nosZI* gene at 80% WFPS (Fig. 3D), and provoked an increase in the bacterial abundance of both clades of *nosZ* at the end of the experiment (Fig. 5B,D). This induction was not observed in other denitrification pathway genes, since the gene expression and gene copy number of *narG*, *nirK* and *nirS* did not vary with the addition of NIs (Supplementary Figures 2, 5). Recent studies have concluded that one-third of all denitrifiers lack *nosZ* and their abundance is affected by different soil properties^32,49^. Moreover, the increase in the ((*nosZI* + *nosZII*)/(*nirK* + *nirS*)) ratio (Fig. 5E,F) suggests specific induction of N_2_O reduction to N_2_ in soils treated with DMPP or DMPSA, which must contribute to the reduction in N_2_O emissions observed after the application of both NIs (Fig. 1). Indeed, we found that *nosZI* gene abundance were negatively correlated with N_2_O emissions (r = −0.373, p < 0.05). This specificity in promoting N_2_O reduction to N_2_ after adding DMPP or DMPSA at 80% WFPS was confirmed by means of a complementary denitrification assay (Fig. 6). Several studies have proposed that elevated NO_3_^−^ content increases the N_2_O:N_2_ ratio^50^ and the effect of NIs on denitrification is indirect, probably due to the shortage of NO_3_^−^^24,30,51^. In contrast, Barrena *et al*.^25^ speculated that DMPP may reduce N_2_O emissions by inducing either gene expression or Nos activity. In agreement with that, in our denitrification assay, which provided the same NO_3_^−^ rate in all treatments, the reason for the increased N_2_O reduction to N_2_ must have been a direct effect of the NIs. Therefore, it appears that NIs have an alternative effect on denitrification that provokes a transient induction of *nosZ* expression (Fig. 3D), which finally stimulates the complete reduction of N_2_O to N_2_ through the action of Nos (Fig. 6). Interestingly, the increase in the ((*nosZI* + *nosZII*)/(*nirK* + *nirS*)) ratio was lower with DMPSA than with DMPP (Fig. 5) and this was in complete agreement with the lower efficiency of DMPSA compared to DMPP in mitigating N_2_O emissions at 80% WFPS (Fig. 1D). In line with our results, Hatch *et al*.^47^ observed that N_2_O production decreased during anaerobic soil incubation with DMPP, concomitant with an increase in N_2_ production, compared to non-DMPP-treated soils.

Interestingly, the action of other types of soil amendments with the capacity to reduce N_2_O emissions, such as biochar, has also been related to a rapid and transient induction of *nosZ* gene expression^46^. Overall, our results evidence the fact that the decrease in N_2_O emissions from NI-treated soils at 80% WFPS is not only caused by nitrification inhibition but also by the stimulation of N_2_O reduction to N_2_ by nitrous oxide reductase during the denitrification process. Our results therefore lead the way towards future studies on the mechanisms underlying the direct effect of DMP-based NIs over nitrous oxide reductase enzymes and *nosZ* gene induction. On the other hand, although in presence of acetylene the differences found after NIs addition were much higher than in non-acetylene-treated bottles (Fig. 6A,B), DMPSA showed a significant reduction in the (N_2_O + N_2_) production level. Therefore, this result suggests a potential specific effect of DMPSA on previous denitrification steps that worth to be also explored in future studies.

To our knowledge, this work is the first microcosm study using DMPSA and the first description of the effect of DMPSA on populations of soil microbes. As stated above, we observed that DMPSA and DMPP behaved differently under 80% WFPS conditions. Both molecules are structurally similar and it is difficult to comprehend why the presence of a phosphate compared to a succinic group should have this kind of impact on inhibitor efficiency. In this sense, further work focusing on the mechanism of action of these NIs is essential to elucidate how DMPSA and DMPP behave in the soil.

## Methods

### Soil sampling and experiment setup

Soil was collected in June 2014, from a 0–30 cm layer of clay loam soil in a wheat field (Table 1), in the Basque Country (Spain). In the laboratory, any roots and stones were removed and the soil was passed through a 2 mm sieve. After this, it was air-dried, homogenised and kept at 4 °C until the start of the experiment. In order to reactivate the soil microorganisms, fourteen days prior to the onset of treatments, the soil was rehydrated with deionised water up to 10% below the final water filled pore space (WFPS) and activated by adding 500 mg of carbon in the form of glucose, and 3 mg of NH_4_NO_3_ per kg of dry soil (equivalent to 10 kg N ha^−1^)^23,52^. The experiment was set up as a soil microcosm incubation study. 272 1 litre glass flasks were prepared with 300 g of dried soil per flask; 3 technical replicates per treatment and time point were sampled destructively for mineral N and pH determinations (a total of 240 bottles), and the remaining 32 flasks were used for N_2_O emissions and soil nitrifying and denitrifying bacterial population analyses (4 technical replicates per treatment). The trial was designed as a split plot arrangement in which eight treatments were established as a result of combining the different soil water content and fertilisers. The treatments were: unfertilised control (C); ammonium sulphate (AS); AS + DMPP (DMPP); and AS + DMPSA (DMPSA). Ammonium sulphate [(NH_4_)_2_SO_4_] was applied at a rate of 42.8 mg N kg^−1^ dry soil (equivalent to 140 kg N ha^−1^); DMPP and DMPSA (EuroChem Agro Iberia S.L.) were both added at 1% of applied N. In order to achieve a homogeneous distribution of the fertilisers in the soil, the AS (with or without inhibitor) was dissolved in deionised water, and subsequently 5 ml were added to the corresponding treatments. For unfertilised treatments, 5 ml of deionised water were added. Each treatment was then subdivided into two sub-treatments with different moisture conditions expressed as water filled pore space (WFPS 40% and 80%). Water was added to every flask in order to reach the humidity defined for each soil water content according to the equation by Aulakh *et al*.^53^: [(gravimetric water content X soil bulk density)/total soil porosity], where soil porosity = [1 — (soil bulk density/particle density)], soil bulk density = 1.14 g cm^−3^, and particle density is assumed to be 2.65 g cm^−3^. In order to maintain the humidity while allowing gas diffusion, the flasks were covered with Parafilm (Oshkosh, WI, USA) throughout the entire study. Twice per week each flask was weighed to check the soil water content, deionised water being added whenever necessary. The microcosms were incubated in the dark at 20 °C throughout the 51 days of the experimental period.

**Table 1:** Physical and chemical properties of the soil (0–30 cm depth).

Soil texture	Sand (%)	36
	Silt (%)	28
	Clay (%)	36
Soil chemical properties	pH	8,4
	OM (%)	2,9
	N (%)	0,23
	C:N	7,31
	Carbonate (%)	2,01
	P (ppm)	106
	Ca (ppm)	1295
	Mg (ppm)	171,4
	K (ppm)	516

Sieved and homogenized soil was used as a single pool to set up the experiment. OM means organic matter.

### N_2_O emissions measurement

Daily N_2_O emissions were determined every two days for the first 16 days, as well as on days 31 and 51. To do this, four independent flasks for each microcosm treatment were closed hermetically and 20 ml of gas from the atmosphere of the hermetic flasks were sampled after 30, 60 and 90 minutes, and stored at pressure in 12 ml vials for later N_2_O analysis. Emission rates were calculated taking into account the increased concentration of N_2_O during the 90 minutes of incubation. The gas samples were analysed an Agilent 7890 A gas chromatograph (GC; Agilent Technologies, Santa Clara, CA, USA) equipped with an electron-capture detector (ECD). The gas samples were injected into a capillary column (IA KRCIAES 6017: 240 °C, 30 m × 320 µm) by means of a headspace auto-sampler (Teledyne Tekmar HT3, Mason, OH, USA) connected to the GC. On every measurement day, N_2_O standards were analysed as internal controls. Cumulative N_2_O production throughout the entire experiment was calculated by multiplying the length of time between two measurements by the average emissions rate for that period, and adding that amount to the previously accumulated N_2_O.

### Geochemical analyses

In order to monitor soil pH and mineral nitrogen (NH_4_^+^ and NO_3_^−^), three samples per treatment and time point were sampled, each from an independent flask. Soil pH is a key factor affecting biological processes as well as the diversity and structure of bacterial populations^54^, and the addition of DMPP may affect this pH^55^. For this reason, we monitored the evolution of soil pH throughout the entire incubation period. To determine soil pH, 10 g of dry soil were suspended in deionised water (1:2, w:v) and shaken for an hour at 165 rpm (KS501D, IKA, Staufen, Germany) to properly homogenise the mixture. Soil suspensions were left to settle for 30 min, to decant the particles, and the pH was determined from the solution. No significant differences were observed between the fertilised treatments (Supplementary Figure 1).

To analyse soil mineral nitrogen, 100 g of dry soil were mixed with 1 M KCl (1:2, w:v) and shaken for an hour at 165 rpm to properly homogenise the mixture. This soil solution was filtered twice; first through Whatman no. 1 filter paper (GE Healthcare, Little Chalfont, Buckinghamshire, UK), and then through Sep-Pak Classic C18 Cartridges 125 Å pore size (Waters, Milford, MA, USA) to eliminate organic carbon. The filtered soil solution was used to determine the NO_3_^−^ content, as described by Cawse^56^, and NH_4_^+^ content using the Berthelot method^57^.

### Nucleic acid isolation

Ten grams of soil were collected from the same flasks as used for N_2_O determination on each measurement day, immediately frozen in liquid nitrogen and stored at −80 °C until use. To quantify bacterial populations, DNA was extracted from 0.25 g of soil using the PowerSoil DNA Isolation Kit (MO BIO Laboratories, Carlsbad, USA) following the manufacturer’s recommendations. DNA was quantified spectophotometrically (Nanodrop, Thermo Scientific, Walthan, MA, USA). For total RNA isolation, 1.5 g of frozen soil was extracted with a RNA PowerSoil Total RNA Isolation Kit following the manufacturer’s protocol (MO BIO Laboratories, Carlsbad, USA). The quantity of RNA was determined spectrophotometrically using a NanoDrop (Thermo Scientific), and the RNA was quality checked with a Bioanalyzer 2100 (Agilent Technologies). For each sample, 100 ng of RNA were retrotranscribed into complementary DNA using the PrimeScript™ RT reagent Kit (Takara-Bio Inc., Otsu, Shiga, Japan). The absence of contamination with genomic DNA was tested in all RNA samples by PCR using 16*S rRNA* gene primers.

### Quantification of nitrifying and denitrifying gene abundance and expression analysis using qPCR

Total bacterial and archaeal abundances (16*S rRNA*), and genes involved in nitrification (*amoA*) and denitrification (*narG*, *nirK, nirS*, *nosZI* and *nosZII)*, were amplified by qPCR using SYBR® *Premix Ex Taq*™ II (Takara-Bio Inc.) using StepOnePlus™ Real-Time PCR System and StepOnePlus™ Software v2.3 (Thermo Scientific). Detailed information about gene-specific qPCR primers, thermal programs and plasmid standard efficiencies are refereed in Supplementary Table 1. Standard curves were prepared from serial dilutions of 10^7^ to 10^2^ gene copies µl^−1^ linearised p-GEMT plasmids with insertions of target gene fragments (Promega Corporation, Madison, WI, USA), following the equations detailed in Correa-Galeote *et al*.^58^. The copy number of target genes per gram of dry soil was calculated from the equation: [(number of target gene copies per reaction X volume of DNA extracted)/(volume of DNA used per reaction X gram of dry soil extracted)] described in Behrens *et al*.^59^. To determine gene expression levels, the same primers and PCR programs were used (Supplementary Table 1). Target gene expression was quantified relative to 16*S rRNA* gene expression calculated with the 2^−∆∆Ct^ method, using the unfertilised soil as calibrator.

### Denitrification assay

In order to determine the effect of both NIs on the nitrous oxide reductase activity (Nos activity), 100 g of dried soil were loaded into 500 ml bottles. The treatments applied were: potassium nitrate (KNO_3_), KNO_3_ + DMPP, and KNO_3_ + DMPSA. In order to favour the denitrification, KNO_3_ was applied at a high rate of 300 mg N kg^−1^ dry soil, NIs were added at 1% of N applied, glucose was added at a rate of 180 mg Kg^−1^ dry soil and the humidity was adjusted to 80% WFPS. The bottles were maintained in the dark at 20 °C and measurements were made 0, 24, and 48 hours after fertilisation. At each time point, 8 bottles per treatment were closed hermetically with rubber septa (Sigma-Aldrich, Inc, USA) and the inner atmospheric headspace was evacuated and fluxed with N_2_ three consecutive times to create an anoxic environment and thus, impel denitrification. To inhibit Nos activity, in four bottles per treatment 10% of the atmosphere was replaced with acetylene (C_2_H_2_)^60^. Then, 5 ml of gas from the headspace of each bottle, either with or without added C_2_H_2,_ were sampled 30, 60 and 90 min after the C_2_H_2_ had been added. Finally, the samples were measured by GC, as detailed previously. The N_2_O production throughout the entire experiment was represented as cumulative emission of N_2_O.

### Statistical analyses

The data was analysed using the IBM SPSS statistics 22 software (Armonk, NY, USA). Normality and homogeneity of variance were analysed using the Kolmogorov-Smirnov and Levene tests. Analysis of significant differences in daily N_2_O emissions, mineral nitrogen, and gene expression levels was carried out by comparison of means (t-test). For bacterial gene copy number, N_2_O cumulative emissions and denitrification assay, significant differences between treatments were analysed using one-way ANOVA with a Duncan post hoc test. Additional details and significance levels are described in the figure captions.

## Electronic supplementary material

Supplementary Information

Dataset 1
