# Supplementary material for: Dimethyl pyrazol-based nitrification inhibitors effect on nitrifying and denitrifying bacteria to mitigate N2O emission
Source: Sci Rep. 2017 Oct 23;7:13810. doi: 10.1038/s41598-017-14225-y (PMC5653738; doi:10.1038/s41598-017-14225-y)
Supplement: Supplementary file 1 — Supplementary Information [file 41598_2017_14225_MOESM1_ESM.pdf]

Supplementary Information

Dimethyl pyrazol-based nitrification inhibitors effect on nitrifying and denitrifying bacteria to mitigate N_2O emission

Fernando Torralbo[1*], Sergio Menéndez[1], Iskander Barrena[1], José M. Estavillo[1], Daniel Marino[1,2] and Carmen González-Murua[1]

Supplementary Table 1. Primers pairs and thermal conditions used for real-time qPCR.

Target group	Primer name	Sequence	Thermal profile	bp length	Efficiency (%)	References
16S rRNA Bacteria	341F	5´-CCTACGGGAGGCAGCAG-3´	95ºC for 2 min – x 1 cycle	174	95	Lopez-Gutiérrez *et al.*, (2004)
	534R	5´-ATTACCGCGGCTGCTGGCA-3´	95ºC for 15 sec , 60 °C for 30 sec , 72 °C for 30 sec , 80 °C for 30sec – x 40 cycles			
16S rRNA Archaea	771F	5´-ACGGTGAGGGATGAAAGCT-3´	95 °C for 2 min – x 1 cycle	226	93	Ochsenreiter *et al.*, (2003)
	957R	5´ -CGGCGTTGACTCCAATTG-3´	95 °C for 15 sec , 58 °C for 30 sec , 72 °C for 30 sec , 80 °C for 30sec – x 40 cycles			
Bacterial *amoA*	amoA1F	5´-GGGGTTTCTACTGGTGGT-3´	95 °C for 2 min – x 1 cycle	491	91	Rotthauwe *et al.*, (1997)
	amoA2R	5´-CCCTCKGSAAAGCCTTCTTC-3´	95 °C for 15 sec, 54 °C for 60 sec , 72 °C for 60 sec – x 40 cycles			
Archaea *amoA*	Arch-amoAF	5´-STAATGGTCTGGCTTAGACG-3´	95 °C for 2 min - x 1 cycle	635	86	Francis *et al.*, (2005)
	Arch-amoAR	5´-GCGGCCATCCATCTGTATGT-3´	95 °C for 45 sec, 54 °C for 45 sec, 72 °C for 45 sec; 85 °C for 20 sec - x 40 cycles			
narG	NarG-f	5'-TCGCCSATYCCGGCSATGTC-3'	95 °C for 2 min – x 1 cycle	173	97	Bru *et al.*, (2007)
	NarG-r	5'-GAGTTGTACCAGTCRGCSGAYTCSG-3'	95 °C for 15 sec, 63 °C for 30 sec (-1 °C /cycle), 72 °C for 30 sec, 80 °C for 30 sec – x 6 cycles			
			95 °C for 15 sec, 58 °C for 30 sec, 72 °C for 30 sec, 80 °C for 30sec – x 40 cycles			
nirS	cd3aF	5´-GTSAACGTSAAGGARACSGG-3 ´	95 °C for 2 min - x 1 cycle	410	92	Michotey *et al.*, (2000)
	R3cd	5´-GASTTCGGRTGSGTCTTGA-3´	95 °C for 45 sec, 55 °C for 45 sec, 72 °C for 45 sec; 85 °C for 20 sec - x 40 cycles			Throbäck *et al.*, (2004)
nirK	NirK 876	5´-ATYGGCGGVCAYGGCGA-3´	95 °C for 2 min – x 1 cycle	165	86	Henry *et al.*, (2004)
			95 °C for 15 sec, 63 °C for 30 sec (-1 °C /cycle), 72 °C for 30 sec, 80 °C for 15 sec – x 6 cycles			
	NirK1040	5´-GCCTCGATCAGRTTRTGGTT-3´	95 °C for 15 sec, 58 °C for 30 sec, 72 °C for 30 sec, 80 °C for 30sec – x 40 cycles			
nosZI	nosZ-F	5´-CGCRACGGCAASAAGGTSMSSGT-3´	95 °C for 2 min – x 1 cycle	267	88	Henry *et al.*, (2006)
			95 °C for 15 sec, 65 °C for 30 sec (-1 °C /cycle), 72 °C for 30 sec, 80°C for 30 sec – x 6 cycles			
	nosZ-R	5´-CAKRTGCAKSGCRTGGCAGAA-3´	95 °C for 15 sec, 60 °C for 30 sec, 72 °C for 30 sec, 80 °C for 30sec – x 40 cycles			
nosZII	nosZ-II-F	5´-CTIGGICCIYTKCAYAC-3´	95 °C for 2 min – x 1 cycle	698	79	Jones *et al.*, (2013)
	nosZ-II-R	5´-GCIGARCARAAITCBGTRC-3´	95 °C for 30 sec, 54 °C for 30 sec, 72 °C for 40 sec, 85 °C for 15 sec - x 40 cycles			

Supplementary Figure 1. Evolution of soil pH at 40% WFPS (A) and 80% WFPS (B) during the whole experiment.

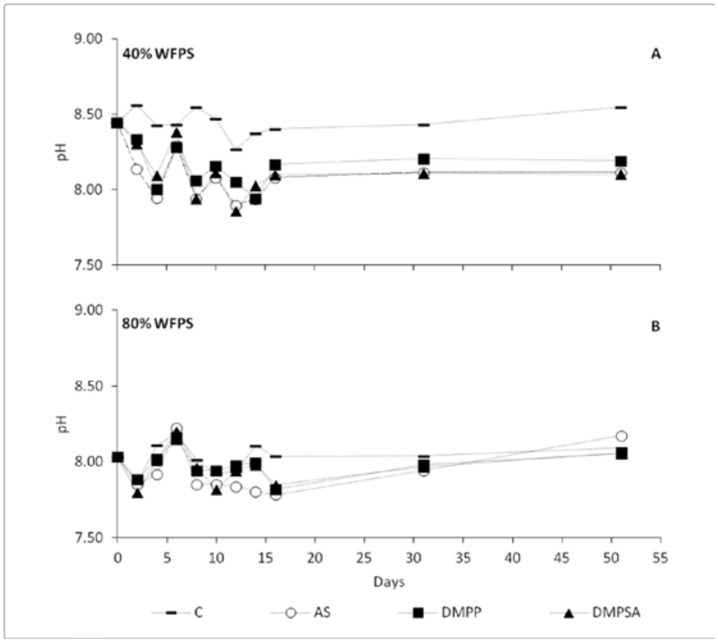

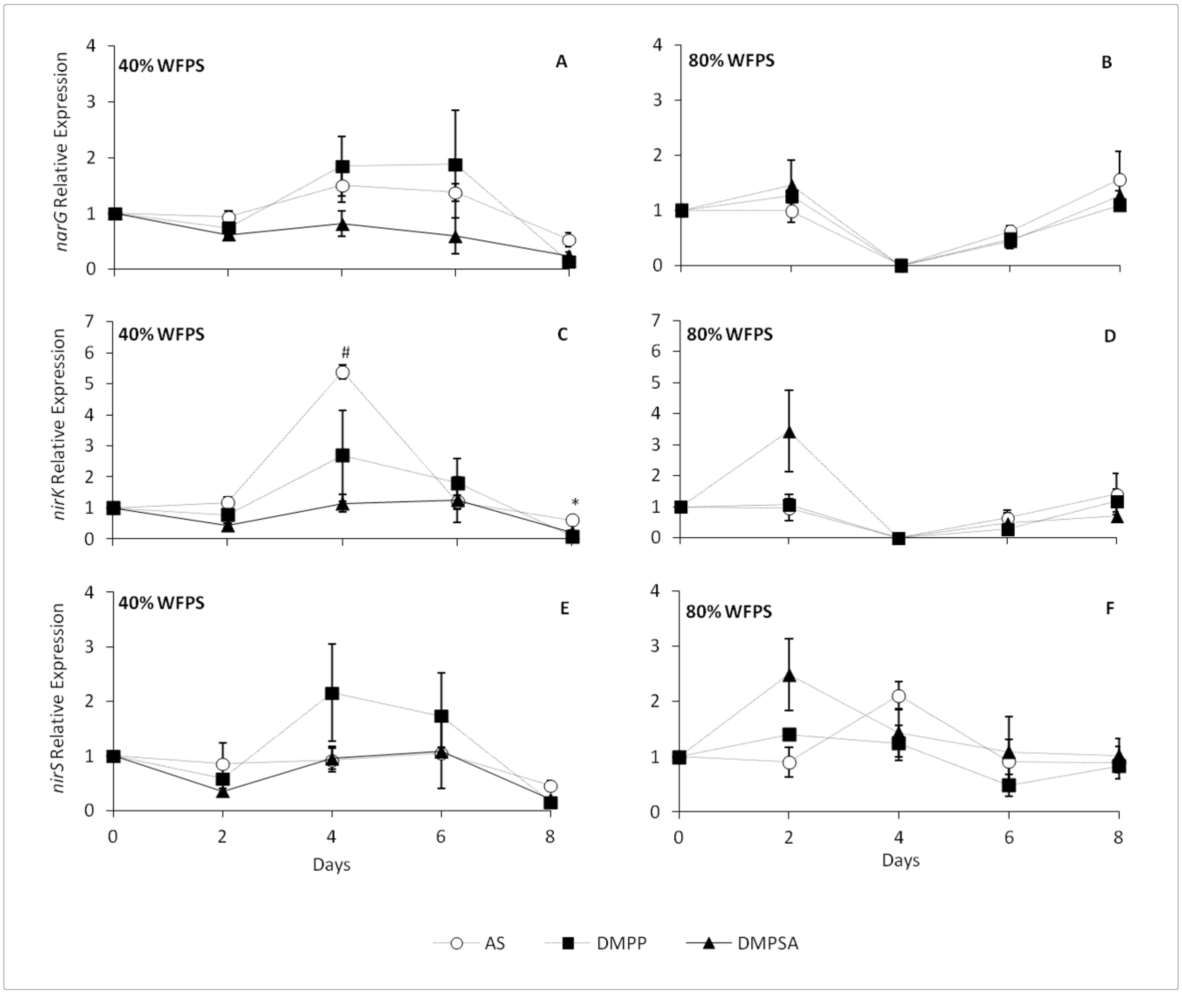

Supplementary Figure 2. Relative expression of denitrifying genes *narG* (A, B), *nirK* (C, D) and *nirS* (E, F) at 40% WFPS (A, C, E) and 80% WFPS (B, D, F) for the first 8 days. Significant differences ($p<0.05$) between DMPP and DMPSA with respect to AS are represented by * and #, respectively. Values represent mean ± SE (n=3).

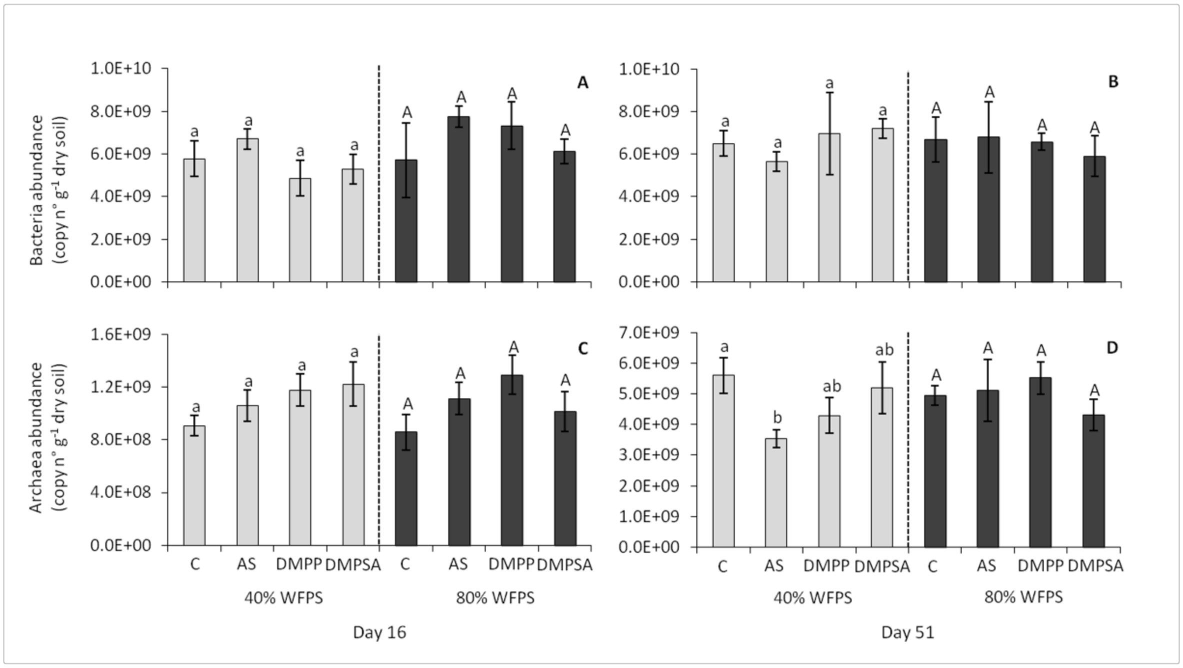

Supplementary Figure 3. Bacteria (A, B) and archaea (C, D) abundances expressed as *16S rRNA* gene copy number per gram of dry soil at 40% WFPS (grey bars) and 80% of WFPS (black bars) 16 (A, C) and 51 days (B, D) after treatment application. Significant differences ($p<0.05$) between treatments within each WFPS condition are indicated with different letters. Asterisk (*) indicates significant WFPS effect for each fertilised treatment ($p<0.05$). Values represent the mean ± SE (n=4). C = unfertilised control; AS = ammonium sulphate; DMPP = ammonium sulphate + DMPP; and DMPSA = ammonium sulphate + DMPSA.

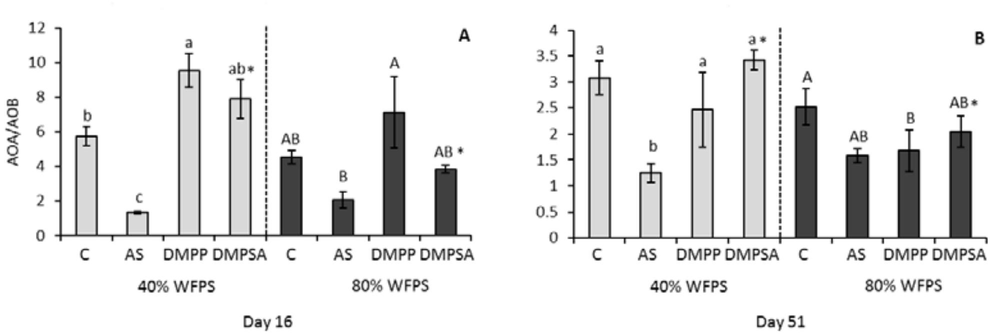

Supplementary Figure 4. Ratio of AOA over AOB at 40% WFPS (grey bars) and 80% of WFPS (black bars) 16 (A, C) and 51 days (B, D) after treatment application. Significant differences ($p<0.05$) between treatments within each WFPS condition are indicated with different letters. Asterisk (*) indicates significant WFPS effect for each fertilised treatment ($p<0.05$). Values represent the mean ± SE (n=4). C = unfertilised control; AS = ammonium sulphate; DMPP = ammonium sulphate + DMPP; and DMPSA = ammonium sulphate + DMPSA.

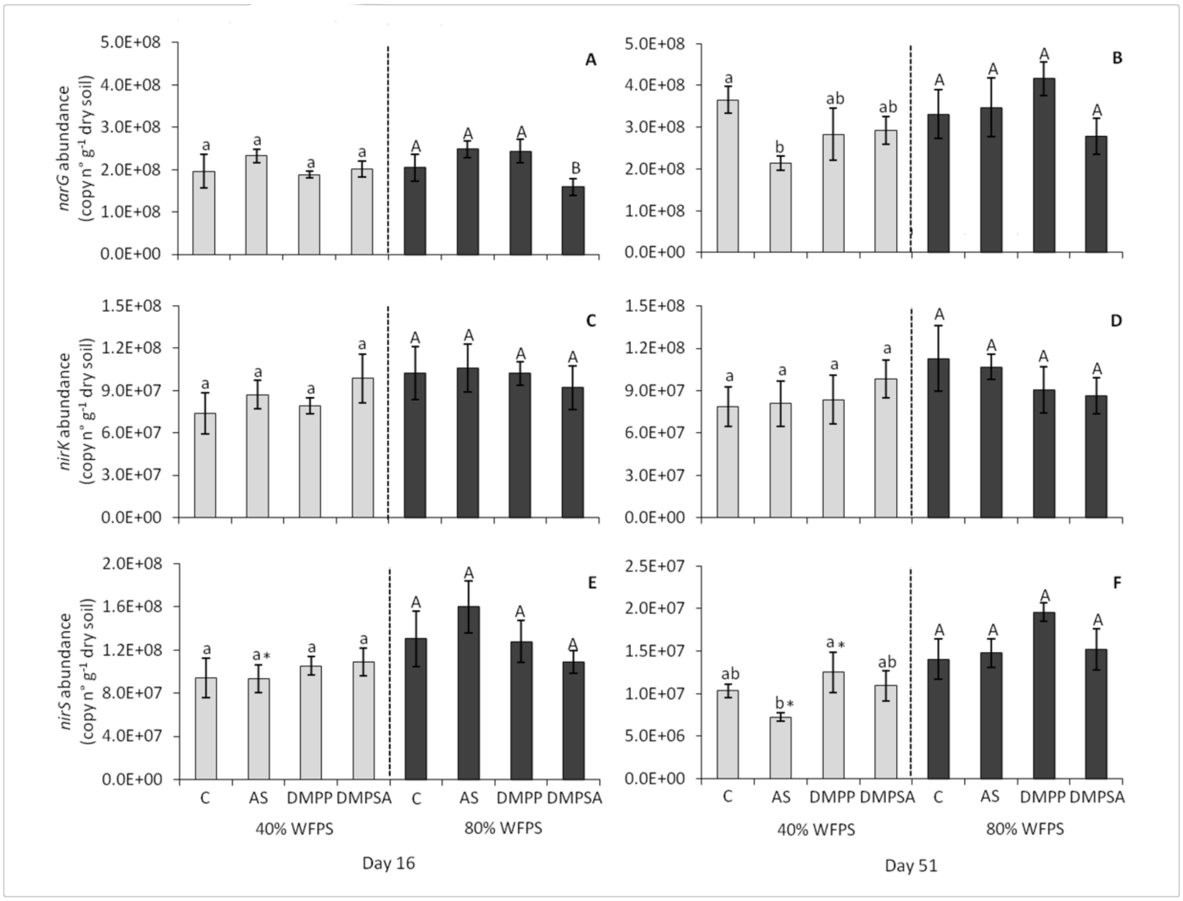

Supplementary Figure 5. Denitrifying abundances expressed as *narG* (A, B), *nirK* (C, D) and *nirS* (E, F) gene copy number per gram of dry soil at 40% WFPS (grey bars) and 80% of WFPS (black bars) 16 (A, C, E) and 51 days (B, D, F) after treatment application. Significant differences ($P < 0.05$) between treatments within each WFPS condition are indicated with different letters. Asterisk (*) indicates significant WFPS effect for each fertilization treatment ($P < 0.05$). Values represent mean ± SE (n=4). C means unfertilized control, AS ammonium sulphate, DMPP ammonium sulphate + DMPP and DMPSA ammonium sulphate + DMPSA.

Supplementary references

1. López-Gutiérrez, J. C. *et al.* Quantification of a novel group of nitrate-reducing bacteria in the environment by real-time PCR. *J. Microbiol. Methods* **57,** 399–407 (2004).
2. Ochsenreiter, T., Selezi, D., Quaiser, A., Bonch-Osmolovskaya, L. & Schleper, C. Diversity and abundance of Crenarchaeota in terrestrial habitats studied by 16S RNA surveys and real time PCR. *Environ. Microbiol.* **5,** 787–797 (2003).
3. Rotthauwe, J. H., Witzel, K. P. & Liesack, W. The ammonia monooxygenase structural gene amoa as a functional marker: Molecular fine-scale analysis of natural ammonia-oxidizing populations. *Appl. Environ. Microbiol.* **63,** 4704–4712 (1997).
4. Francis, C. a, Roberts, K. J., Beman, J. M., Santoro, A. E. & Oakley, B. B. Ubiquity and diversity of ammonia-oxidizing archaea in water columns and sediments of the ocean. *Proc. Natl. Acad. Sci. U. S. A.* **102,** 14683–14688 (2005).
5. Bru, D., Sarr, A. & Philippot, L. Relative abundances of proteobacterial membrane-bound and periplasmic nitrate reductases in selected environments. *Appl. Environ. Microbiol.* **73,** 5971–5974 (2007).
6. Michotey, V., Méjean, V. & Bonin, P. Comparison of methods for quantification of cytochrome cd_1- Denitrifying bacteria in environmental marine samples. *Appl. Environ. Microbiol.* **66,** 1564–1571 (2000).
7. Throbäck, I. N., Enwall, K., Jarvis, Å. & Hallin, S. Reassessing PCR primers targeting *nirS*, *nirK* and *nosZ* genes for community surveys of denitrifying bacteria with DGGE. *FEMS Microbiol. Ecol.* **49,** 401–417 (2004).
8. Henry, S. *et al.* Quantification of denitrifying bacteria in soils by *nirK* gene targeted real-time PCR. *J. Microbiol. Methods* **59,** 327–335 (2004).
9. Henry, S., Bru, D., Stres, B., Hallet, S. & Philippot, L. Quantitative detection of the *nosZ*

gene, encoding nitrous oxide reductase, and comparison of the abundances of 16S rRNA, *narG*, *nirK*, and *nosZ* genes in soils. *Appl. Environ. Microbiol.* **72,** 5181–5189 (2006).

10. Jones, C. M., Graf, D. R. H., Bru, D., Philippot, L. & Hallin, S. The unaccounted yet abundant nitrous oxide-reducing microbial community: a potential nitrous oxide sink. *ISME J.* **7,** 417–26 (2013).
